# Supplementary material for: Genetic Preference for Sweet Taste in Mothers Associates with Mother-Child Preference and Intake
Source: Nutrients. 2023 May 30;15(11):2565. doi: 10.3390/nu15112565 (PMC10255080; doi:10.3390/nu15112565)

Supplementary Figure S2. Forest plot illustrating associations between single SNPs in additive (AD), major (D1), or minor (D2) dominant models with the outcomes (**A**) sweet preference score, or (**B**) sweet intakes per week by FFQ1, and (**C**) sweet intakes per week by FFQ2 among 187 mothers who donated saliva for DNA extraction.

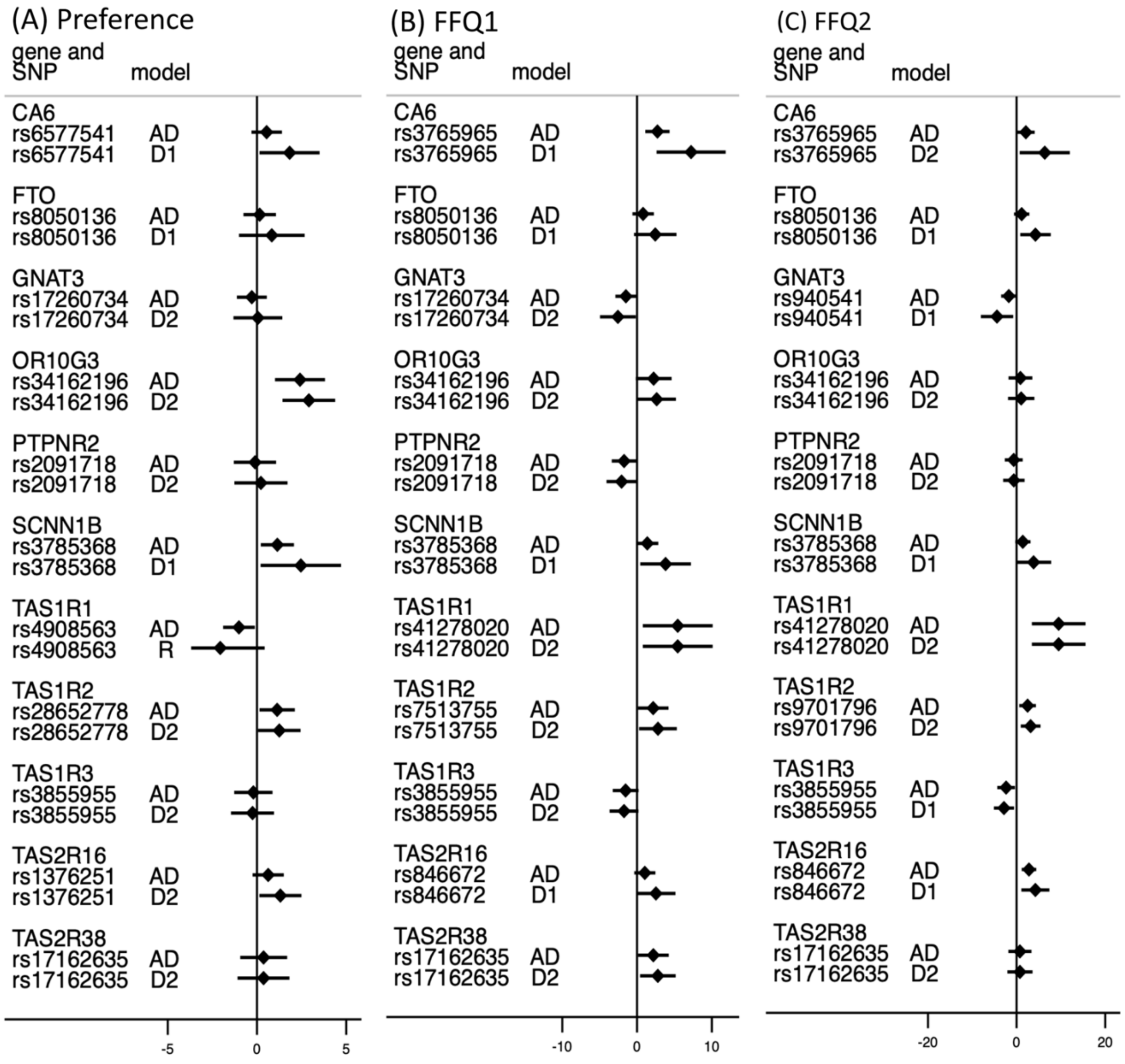

Supplement: Supplementary file 1 [file nutrients-15-02565-s001.zip › 2_Supplementary Figure S2.pdf]
