# Supplementary figures and images for: Genetic Preference for Sweet Taste in Mothers Associates with Mother-Child Preference and Intake
Source: Nutrients. 2023 May 30;15(11):2565. doi: 10.3390/nu15112565 (PMC10255080; doi:10.3390/nu15112565)

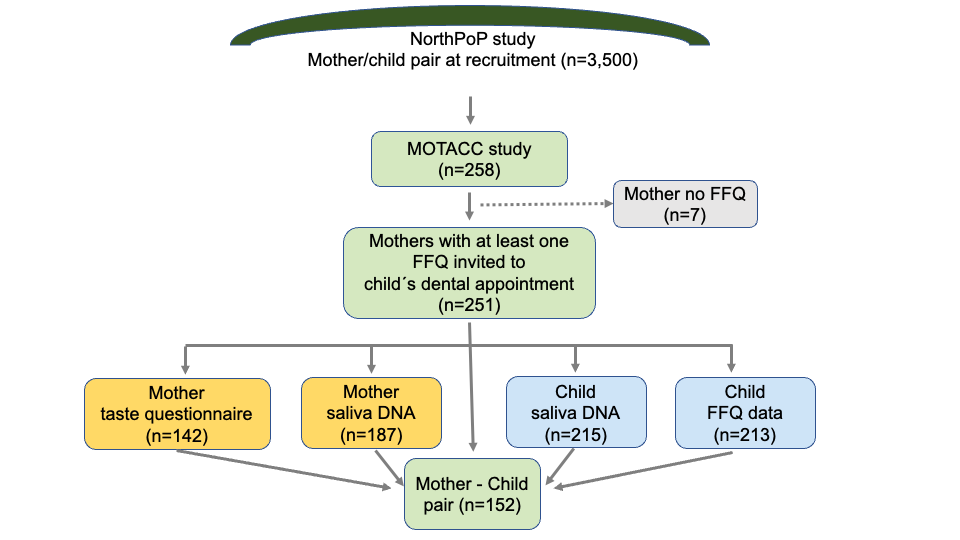

Supplement: Supplementary file 1 [file nutrients-15-02565-s001.zip › 1_Supplementary Figure S1.tiff]
